# Targeted nanoparticles triggered by plaque microenvironment for atherosclerosis treatment through cascade effects of reactive oxygen species scavenging and anti-inflammation

**DOI:** 10.1186/s12951-024-02652-9

**Published:** 2024-07-27

**Authors:** Xianghong Luo, Mengjiao Zhang, Waicong Dai, Xianghao Xiao, Xinyi Li, Yingjian Zhu, Xiangyang Shi, Zhaojun Li

**Affiliations:** 1 Department of Echocardiography, Shanghai General Hospital, School of Medicine, Shanghai Jiao tong University, Shanghai, 200080 China; 2 Department of Medical Imaging, Weifang Medical University, Weifang, 261053 Shandong China; 3 Department of Ultrasound, Shanghai General Hospital, Shanghai Jiao Tong University School of Medicine, Shanghai, 200080 China; 4 State Key Laboratory for Modification of Chemical Fibers and Polymer Materials, Shanghai Engineering Research Center of Nano-Biomaterials and Regenerative Medicine, College of Biological Science and Medical Engineering, Donghua University, Shanghai, 201620 China; 5 Department of Urology, Jiading Branch of Shanghai General Hospital, Shanghai Jiao Tong University School of Medicine, Shanghai, 201803 China; 6 Department of Ultrasound, Jiading Branch of Shanghai General Hospital, Shanghai Jiao tong University School of Medicine, Shanghai, 201803 China; 7 School of Life Sciences, Hubei University, Hubei, China

**Keywords:** Low molecular weight heparin, Lipoic acid, Reactive oxygen species, Drug delivery, Atherosclerosis

## Abstract

Inflammatory factors and reactive oxygen species (ROS) are risk factors for atherosclerosis. Many existing therapies use ROS-sensitive delivery systems to alleviate atherosclerosis, which achieved certain efficacy, but cannot eliminate excessive ROS. Moreover, the potential biological safety concerns of carrier materials through chemical synthesis cannot be ignored. Herein, an amphiphilic low molecular weight heparin- lipoic acid conjugate (LMWH-LA) was used as a ROS-sensitive carrier material, which consisted of injectable drug molecules used clinically, avoiding unknown side effects. LMWH-LA and curcumin (Cur) self-assembled to form LLC nanoparticles (LLC NPs) with LMWH as shell and LA/Cur as core, in which LMWH could target P-selectin on plaque endothelial cells and competitively block the migration of monocytes to endothelial cells to inhibit the origin of ROS and inflammatory factors, and LA could be oxidized to trigger hydrophilic-hydrophobic transformation and accelerate the release of Cur. Cur released within plaques further exerted anti-inflammatory and antioxidant effects, thereby suppressing ROS and inflammatory factors. We used ultrasound imaging, pathology and serum analysis to evaluate the therapeutic effect of nanoparticles on atherosclerotic plaques in apoe^−/−^ mice, and the results showed that LLC showed significant anti-atherosclerotic effects. Our finding provided a promising therapeutic nanomedicine for the treatment of atherosclerosis.

**Supplementary Information:**

The online version contains supplementary material available at 10.1186/s12951-024-02652-9.

## Introduction

Atherosclerosis is a chronic inflammatory disease, accompanied by endothelial damage, monocyte/macrophage adhesion, foam cell formation, plaque rupture, etc. [1–3]. In plaque inflammation, ROS plays a crucial role, which can induce oxidative stress to promote atherosclerosis [4]. Low-density lipoprotein (LDL) oxidation caused by high level of ROS is considered to be the key link in the development of atherosclerosis [5, 6]. Oxidized low-density lipoprotein (ox-LDL) will cause vascular endothelial damage and promote foam cells formation [7, 8]. In turn, damaged endothelial cells will recruit a large number of monocytes, increasing the content of inflammatory cells in the plaque, and the increase of inflammatory cells, including foam cells, will also accelerate the increase of ROS level [9]. This ROS mediated inflammatory feedback regulation will further worsen atherosclerosis. However, clinical therapy for atherosclerosis is still limited to lipid-lowering drugs. As a result, it is urgent to find a simple and effective strategy to attenuate oxidative stress for the treatment of atherosclerosis.

Antioxidants, such as vitamin C [10, 11], vitamin E and its derivatives [12–14], polyunsaturated fatty acid [15, 16] and polyphenols [17, 18], have been investigated in atherosclerosis treatment. Although these preclinical studies have confirmed foods or drugs rich in these antioxidants showed protective effects of antioxidants on atherosclerosis, clinical trials did not afford positive effects [19, 20]. On the one hand, these antioxidants are difficult to achieve long-term retention in the body with appropriate formulations, and rapid liver and kidney clearance makes it difficult to exert their pharmacological effects. Further, lack of precise therapeutic targets leads to low drug delivery efficiency and poor accumulation at atherosclerotic plaques [21]. Moreover, the limited ROS-eliminating efficiency by single antioxidant also contributes to unsatisfactory situation of most existing formulations clinically studied in atherosclerosis [22]. Therefore, new anti-oxidative stress strategies remain to be developed.

Nanoparticle-based targeting strategies have been widely adopted for therapy of atherosclerosis [23–28]. The damaged vascular endothelium in plaques increases its permeability, allowing nanoparticles to aggregate at the plaque site through the enhanced permeability and retention (EPR) effect [29]. Besides, the hydrophilic layer outside the nanoparticles can effectively prevent the clearance of the endothelial reticular system and achieve long-term circulation [30, 31]. Wang et al. established a ROS-eliminating β-cyclodextrin material containing tempol and phenylboronic acid pinacol ester, which exerted its inherent antioxidant and anti-inflammatory effects to inhibit atherosclerosis [20]. Ma et al. developed ROS-responsive nanoplatform containing thioether hydrophobic core for acute and chronic Inflammation therapy. [29] Liu et al. also proposed an integrated cascade nanozyme with superoxide dismutase and glutathione peroxidase-like activities, which provided a cascade therapeutic intervention against oxidative stress in senescent cells for atherosclerosis treatment [32]. Although antioxidant materials have shown good efficacy in the treatment of atherosclerosis, the complex preparation process of nanoparticles and biosafety issues of carrier materials have prevented nanomedicine from entering clinical trials.

**Fig. 1:**
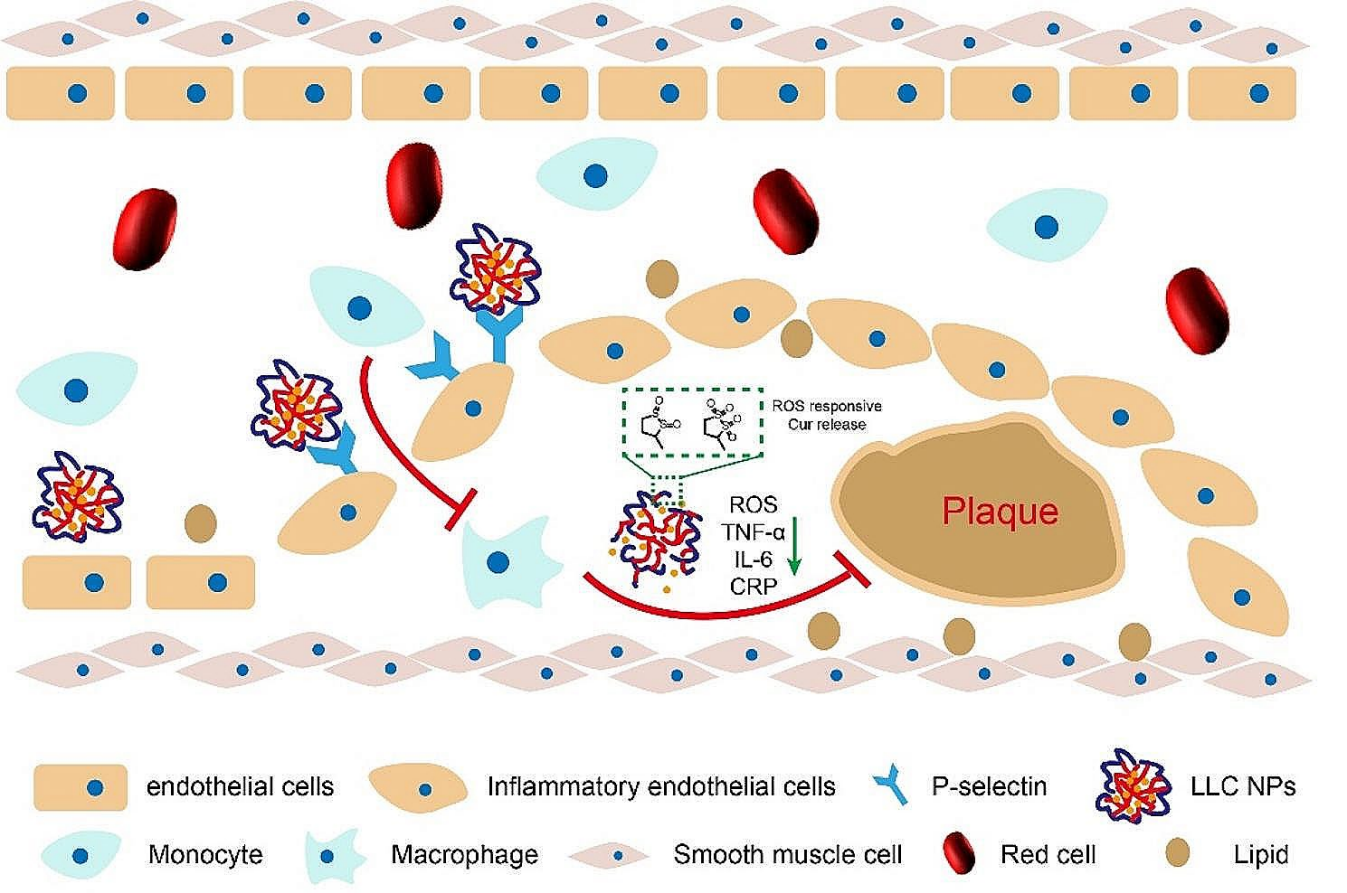
A nanotherapeutic cascade strategy to inhibit plaque inflammation for enhanced anti-atherosclerosis therapy

Herein, we chose low molecular weight heparin (LMWH) and lipoic acid (LA), which have already entered clinical practice, as the backbone of drug delivery systems, avoiding unknown biological toxicity of carrier materials. Our previous research has established that LMWH can bind to P-selectin competitively, inhibiting the recruitment of monocytes and macrophages by endothelial cells, thereby reducing the production source of ROS [33]. The disulfide bond in LA exhibited both antioxidant activity and the properties of ROS triggered hydrophilic-hydrophobic transitions. LA was grafted onto the LMWH chain to form amphiphilic macromolecules, which might encapsulate the hydrophobic anti-inflammatory drug curcumin (Cur) to form stable, ROS sensitive, and biocompatible micelles. Among them, as the outer shell of micelles, LMWH can target overexpressed P-selectin on plaque endothelial cells and block the adhesion of monocytes to vascular endothelium. As the core of micelles, LA can clear excessive ROS in plaques and achieve responsive release of Cur. The released Cur will further exert its anti-inflammatory and antioxidant effects to inhibit atherosclerosis (Fig. 1). This strategy of cascading inhibition of ROS within plaques provided a promising idea for the treatment of atherosclerosis.

## Result and discussion

### Comprehensive characterization of LMWH-LA conjugate and its self-assembled nanoparticles

**Fig. 2:**
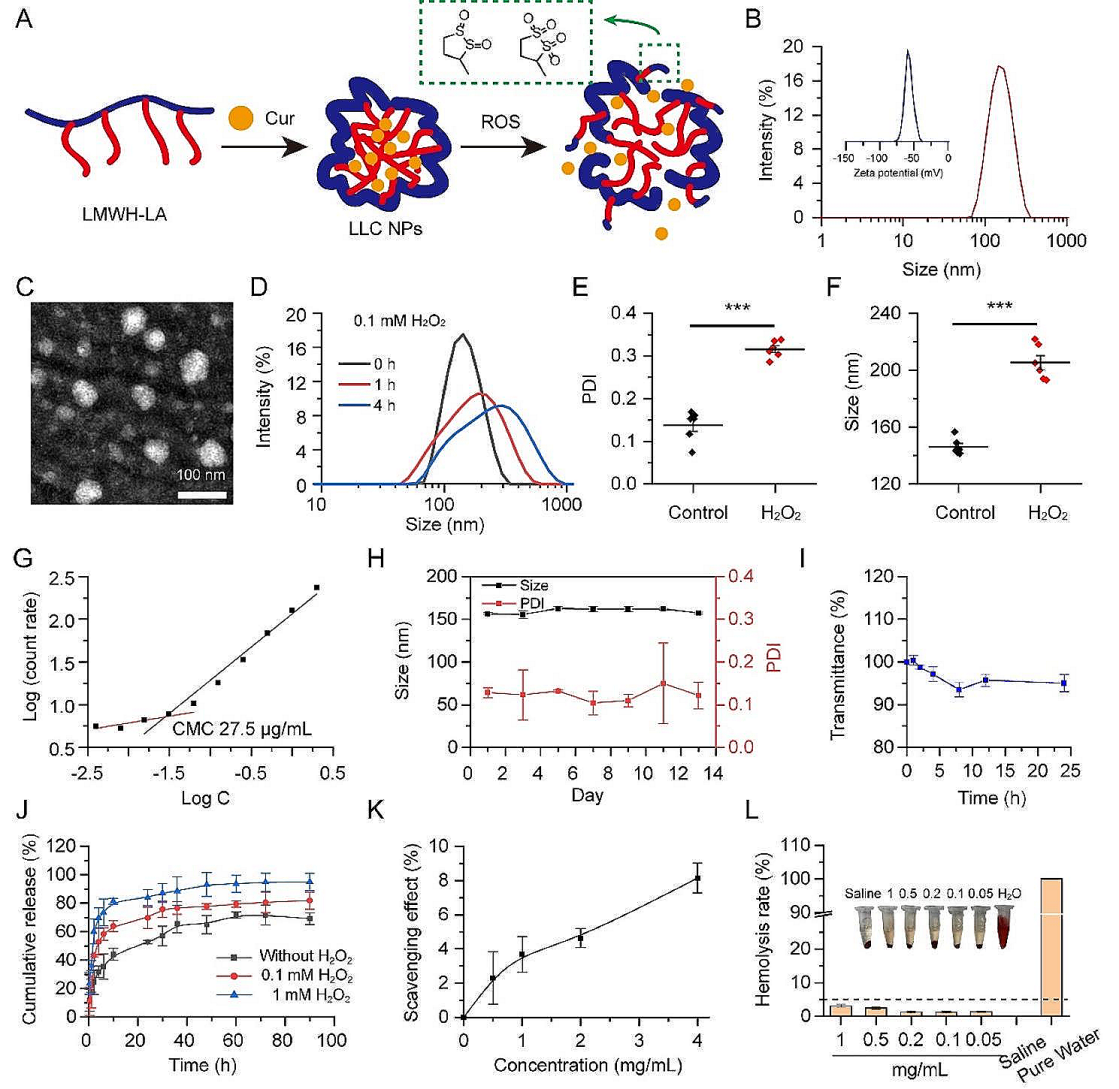
The LMWH-LA conjugates self-assembles into a stable, ROS sensitive micelles with controllable release and nice blood compatibility. (**A**) Schematic diagram of self-assembly and ROS response of LLC NPs. (**B**) Particle size and zeta potential of LLC NPs. (**C**) TEM images of LLC NPs (scale bar = 100 nm). (**D**) Particle size distribution of LL NPs under ROS stimulation for 1 h and 4 h. (**E**, **F**) The changes in PDI and particle size under ROS stimulation for 4 h. means ± SD, *n* = 6. (**G**) The CMC of LL NPs. (**H**) The stability of LLC NPs over a 15-day period at 4 °C. Means ± SD, *n* = 3. (**I**) The stability of NPs in serum for 24 h when incubated with 50% FBS at 37 °C. Means ± SD, *n* = 3. (**J**) Cumulative Cur release of LLC NPs in PBS pH 7.4 under different ROS concentrations. Means ± SD, *n* = 3. (**K**) The ROS scavenging effect of LL NPs. means ± SD, *n* = 3. (**L**) The ratio of hemolysis in red blood cells exposed to LL NPs at different concentrations. Means ± SD, *n* = 3. Embedded are images of red blood cell suspension

Inflammatory cells are involved in many key links in the development of atherosclerosis, such as adhesion of monocytes, polarization of macrophages, and secretion of ROS and inflammatory factors. In our previous studies, nanoparticles with LMWH as shells could competitively bind P-selectin on injured vascular endothelial cells with monocytes, thereby reducing monocytes’ adherence to vascular endothelium [33]. The high ROS level on plaque also guided us to rationally choose LA as a ROS trigger and scavenger. In order to achieve cascade inhibition of plaque inflammation, LA was conjugated to the LMWH chain to form a multifunctional amphipathic drug carrier for atherosclerosis targeted therapy. The hydrophobic LA segment can respond to the high ROS in the plaque and transform into hydrophilic, thereby releasing the encapsulated drugs (Fig. 2A). The chemical structures obtained were analyzed using proton nuclear magnetic resonance ([1]H NMR) spectra and Fourier transform infrared (FT-IR) spectroscopy (Fig. S1-2). Through thermogravimetric analysis (TGA), it was determined that the content of lipoic acid (LA) within the LMWH-LA conjugate was around 20% (w/w) (Fig. S3).

Thanks to their amphiphilic chemical structures, LMWH-LA is capable of self-assembling into micelles, known as LL NPs, featuring the LMWH chain as the hydrophilic outer shell and the LA unit as the hydrophobic inner core. Cur can be encapsulated within these micelles, forming LLC NPs through hydrophobic interactions. Dynamic light scattering (DLS) measurements revealed that the LL NPs have an average size of about 143 nm (Fig. S4). Upon incorporating Cur, the particle size of the LLC NPs expanded to 160 nm (Fig. 2B). The LLC NPs exhibited a negative ζ potential of -50 mV. The loading capacity for Cur and the encapsulation efficiency within the nanoparticles were measured to be 7.8% and 85%, respectively. TEM showed that LLC NPs were spherical in morphology (Fig. 2C). To confirm the ROS sensitivity, the size distribution and polydispersity index (PDI) of LL NPs incubated with 0.1 mM H_2_O_2_ were evaluated. The size significantly increased and the size distribution significantly widened after 4 h stimulation, showing an obvious ROS sensitivity of LL NPs (Fig. 2D, E, F). The good stability of NPs was a prerequisite for entering clinical research. Firstly, the CMC of the LL NPs was evaluated by dynamic light scattering. In Fig. 2G, the CMC was calculated as 27.5 µg/mL. Next, the storage stability of LLC NPs at 4 °C was assessed by monitoring changes in nanoparticle size and PDI, with no notable alterations in either parameter observed over a 13-day period, demonstrating their favorable storage stability (Fig. 2H). Then, the stability of NPs in serum was evaluated by incubating them with 50% fetal bovine serum (FBS) at 37 °C. Over a 24-hour period, there was no significant variation in transmission, indicating that the LLC NPs maintained their stability in 50% FBS (Fig. 2I). Furthermore, the release pattern of the drug from LLC NPs was analyzed in PBS (containing 0, 0.1 and 1 mM H_2_O_2_) by examining the Curcumin release profiles. The presence of H_2_O_2_ would accelerate the release of Cur, and the higher H_2_O_2_ concentration, the faster the drug release, which was related to the change in hydrophobicity of the micelle core (Fig. 2J). Previous studies have pointed out LA was regarded as potent cellular oxidation regulators with extraordinary antioxidant properties [34]. So, it is necessary to explore the antioxidant properties of LA grafted onto LMWH. As shown in Fig. 2K, LL NPs revealed good antioxidant properties, and its antioxidant capacity was positively correlated with the concentration. Moreover, initial results from hemolysis tests indicated that the hemolysis rates of LL NPs did not exceed 5% at a concentration of 1000 µg/mL and the red blood cells showed normal morphology, indicating that LL NPs posed no hemolysis risk. (Fig. 2L, Fig. S5).

Overall, the components of LMWH-LA were all derived from clinical drugs, and its structure was clear. The NPs formed by LMWH-LA have good stability, ROS sensitivity, antioxidant capacity and good hemocompatibility, which provided a basis for subsequent anti-atherosclerosis therapy.

### Suppression of the vascular inflammation cascade through in vitro NPs intervention

**Fig. 3:**
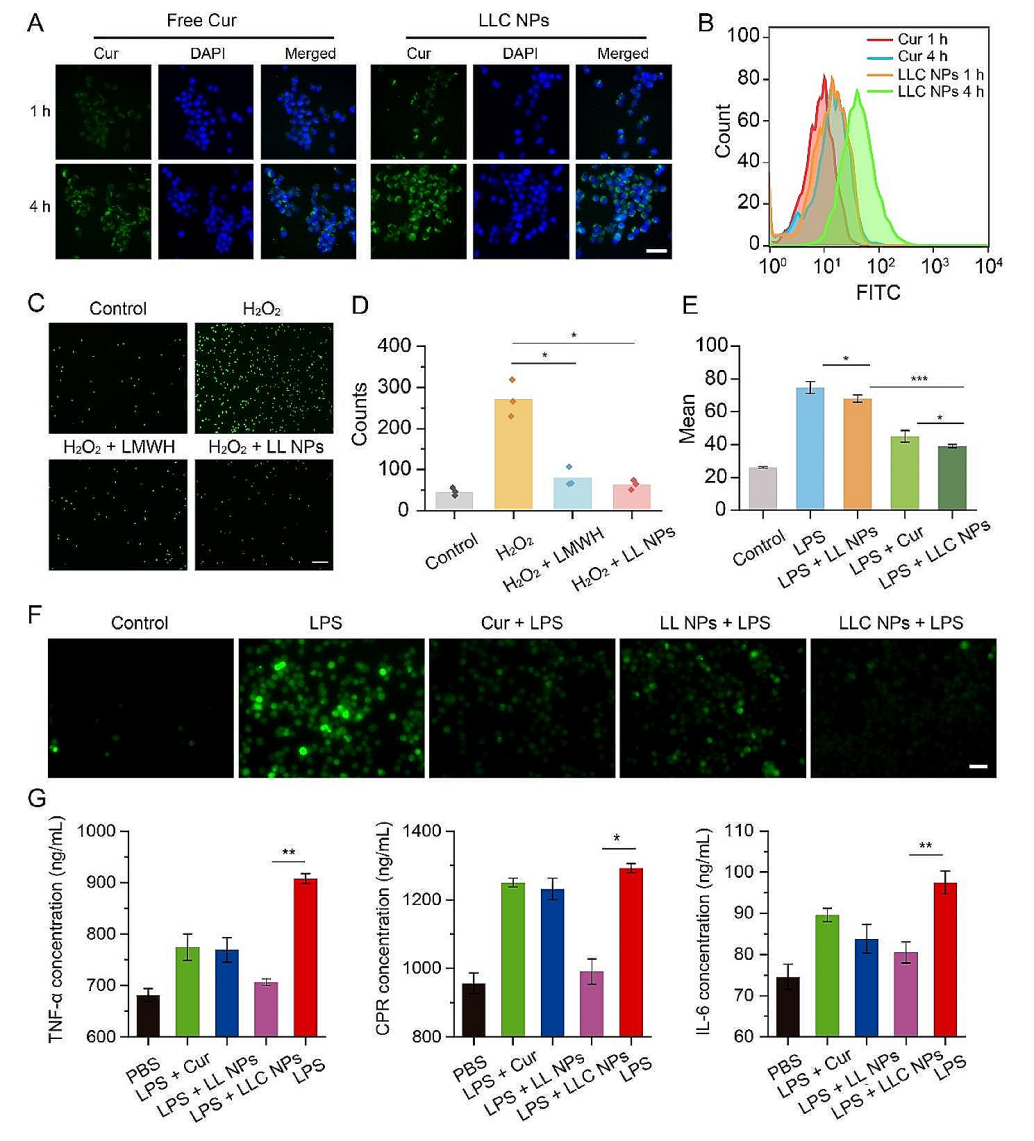
Suppression of the vascular inflammation cascade through in vitro NPs intervention. (**A**, **B**) The uptake of free Cur and LLC NPs by was evaluated by fluorescence microscopy and flow cytometry (Scale bar = 20 μm). (**C**) Images and (**D**) and quantitative assessment of THP-1 cells labeled with green fluorescence adhering to HUVECs, following various treatment. (Scale bar = 20 μm). Mean ± SD, *n* = 3. *P < 0.05. (**E**) Fluorescent imaging revealed ROS production in RAW 264.7 cells exposed to LPS, following their staining with DCFH-DA (Scale bar = 10 μm). (**F**) Flow cytometry was used to quantify the levels of intracellular ROS in RAW264.7 cells following various treatments. Mean ± SD, *n* = 3. *P < 0.05, ***p < 0.001. (**G**) Inflammatory cytokines TNF-α, IL-6, CRP secreted by RAW264.7 cells treated with different formulation. Mean ± SD, *n* = 5. *P < 0.05, **p < 0.01

Macrophages play an important role in plaque inflammation. In this part, the cytotoxicity and cellular uptake of NPs in RAW264.7 cells were investigated through in vitro experiments. LL NPs had no toxicity to RAW264.7 cells in Fig. S6. Figure 3A depicted the cellular uptake. Following a 1-hour incubation period with LLC NPs, green fluorescence was observed surrounding the nucleus of RAW264.7 cells, demonstrating the rapid entry of LLC NPs into RAW264.7 cells. When the co-incubation time reached 4 h, stronger green fluorescence was observed, indicating a time-dependent uptake of NPs by RAW264.7 cells. In contrast, free Cur showed a lower uptake efficiency by RAW264.7 cells due to its poor water solubility. In addition, flow cytometry also revealed that LLC NPs were more easily taken up by cells than free Cur (Fig. 3B).

Monocyte adhesion to compromised endothelial cells within blood vessels marks the initial phase of plaque-related inflammation. Herein, HUVECs were treated with H_2_O_2_ to create an inflammatory vascular endothelial cell model characterized by elevated P-selectin expression, after which the anti-adhesion properties of NPs were assessed. As shown in Fig. 3C, green fluorescence was used to mark monocytes (THP-1 cells). Following incubation with HUVECs treated with H_2_O_2_, significant green fluorescence was observed, indicating that the injured HUVECs had a high level of adhesion to numerous THP-1 cells due to increased P-selectin expression. Conversely, untreated HUVECs showed minimal adhesion of THP-1 cells. The adherence of THP-1 cells decreased when the H_2_O_2_-treated HUVECs were pre-treated with LMWH or LL NPs. Quantitative statistics were conducted on monocytes from any three fields of view in each group, which also indicated that LL NPs demonstrated effective anti-cell adhesion properties (Fig. 3D).

Monocytes adhere to the endothelium of blood vessels and then penetrate into the subintimal layer, polarizing into macrophages. These macrophages then release reactive oxygen species (ROS) and various inflammatory agents, contributing to the progression of inflammation within arterial plaques. Herein, the anti-inflammatory and antioxidant effects of LLC NPs were comprehensively evaluated. Specifically, macrophages (RAW264.7 cells) activated by lipopolysaccharide (LPS) were utilized as an inflammatory cell model, and ROS and the inflammatory factor TNF-α, IL-6, and C-Reactive Protein (CRP) were used as the main indexes. First, ROS production was detected with 2’, 7’-dichlorofluorescein diacetate (DCFH-DA). As shown in Fig. 3F, Under LPS induction, RAW264.7 cells exhibited significant ROS production, visible as green fluorescence, in contrast to the minimal ROS generated by unstimulated macrophages. However, pretreating these cells with LLC NPs effectively reduced ROS levels upon LPS challenge, attributed to the anti-oxidative properties of Cur. In addition, the unloaded-Cur LL NPs also exhibited a certain antioxidant effect due to the presence of disulfide bond. The quantitative analysis of flow cytometry also confirmed the strong antioxidant effect of LLC NPs (Fig. 3E). Then, the inflammatory factor TNF-α, IL-6 and CRP were detected by ELISA. After stimulation with LPS, RAW264.7 cells produced significant amounts of inflammatory factor. However, pretreatment with LLC NPs effectively reduced the secretion of inflammatory factors, as demonstrated in Fig. 3G. Notably, LLC NPs were more efficient than free Cur in decreasing both ROS and inflammatory factors’ production, likely due to Cur’s limited solubility and bioavailability.

### Imaging evaluation of NPs targeted therapy for plaque

**Fig. 4:**
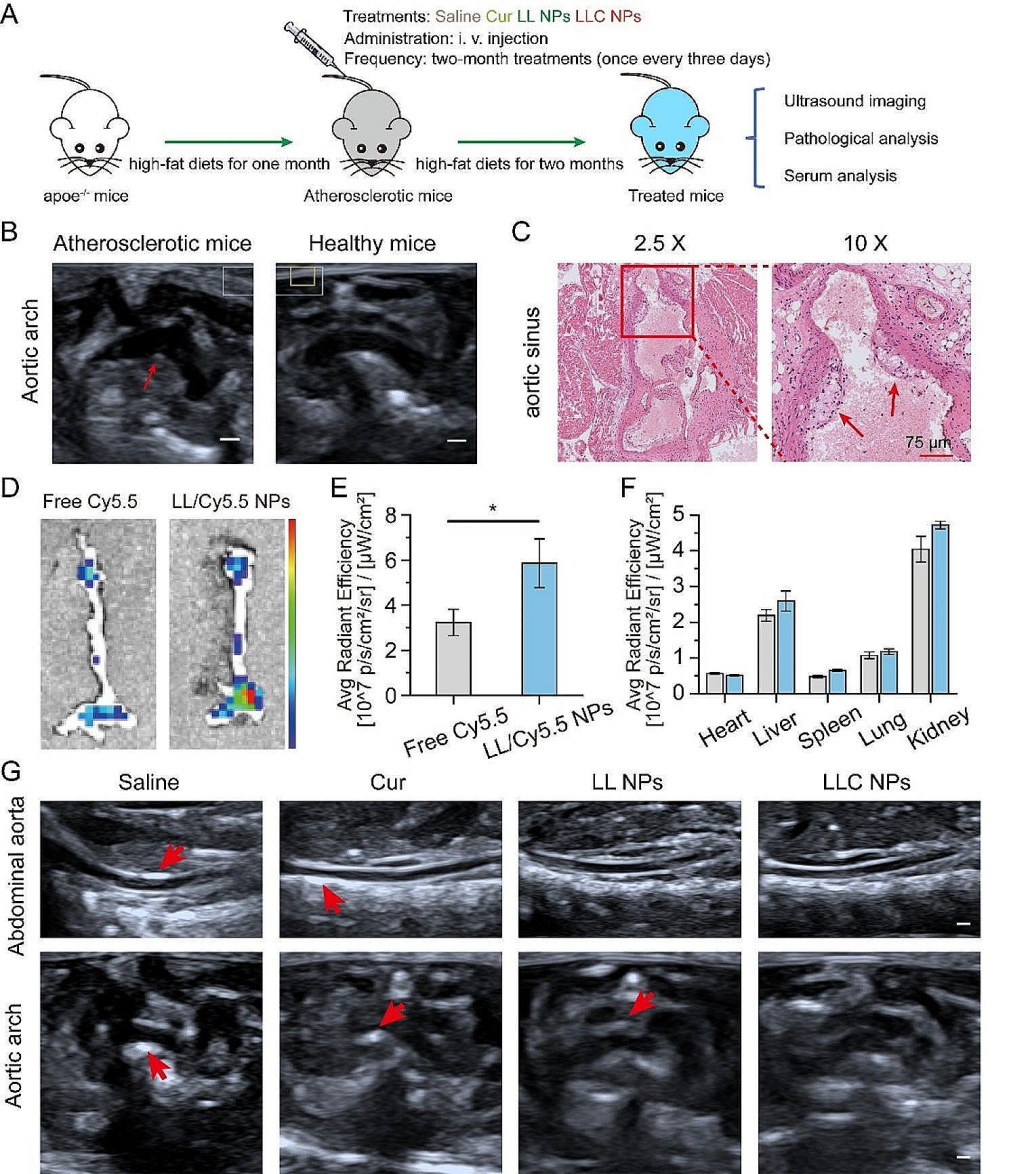
Imaging evaluation of NPs targeted therapy for plaque. (**A**) The treatment plan for atherosclerosis prevention and management. (**B**) Ultrasound imaging of aortic arch in early atherosclerotic mice and healthy mice (Scale bar = 2 mm). (**C**) H&E staining of aortic sinus in early atherosclerotic mice (Scale bar = 75 μm). (**D**) Ex vivo fluorescence images of the isolated aorta. (**E**, **F**) The quantitative average fluorescence intensity measurements for the aorta (**E**) and primary organs (**F**) across all experimental groups. Mean ± SD, *n* = 3, *p < 0.05. (**G**) Ultrasound images of abdominal aorta and aortic arch after treatment (Scale bar = 2 mm)

In view of nice anti-inflammatory effect, the effectiveness of LLC NPs in countering atherosclerosis was assessed in vivo, following the steps outlined in Fig. 4A. Eight-week-old male apoe^−/−^ mice were fed with a high-fat diet for a month. Subsequently, the structure of the aortic arch in mice was examined using ultrasound imaging. As shown in Fig. 4B, the inner wall of the aortic arch displayed a pronounced echo, as indicated by the red arrow, which was the imaging feature of arterial atherosclerosis. The formation of foam cells is the main feature of atherosclerotic plaque. Therefore, the aortic sinus of mice was removed, and H&E staining was conducted. In Fig. 4C, there was a noticeable presence of numerous foam cells in the intima, as shown by the red frame.

Due to damage to the vascular endothelium and subsequent inflammatory reactions, the permeability of the vascular endothelium at areas with plaques increased significantly. This change facilitates the accumulation of nanoparticles (NPs) within the plaques. The ability of NPs to target plaques in vivo was determined through fluorescence imaging of extracted tissues, as depicted in the mentioned Fig. 4D. 24 h after injection, the aortas from apoe^−/−^ mice treated with Cy5.5-labeled LL NPs showed prominent fluorescence signals compared to those receiving free Cy5.5, indicating NPs had gathered within the plaques. Quantitative analysis of the fluorescence further highlighted the superior accumulation of Cy5.5-labeled LL NPs in the aorta over free Cy5.5, underscoring the NPs’ remarkable ability to target plaques effectively (Fig. 4E). Furthermore, significant fluorescence was observed in both the liver and kidneys, indicating these organs primarily process and metabolize NPs (Fig. 4F).

Following the outlined treatment regimen (Fig. 4A), ultrasound imaging of the abdominal aorta and aortic arch revealed post-treatment changes in Fig. 4G. Enhanced echoes, indicating atherosclerotic plaques, were seen in the saline-treated apoe^−/−^ mice, especially along the abdominal aorta’s inner walls. In contrast, mice treated with NPs displayed less echo enhancement, suggesting a reduction in atherosclerosis, with LLC NPs showing notable plaque progression inhibition. Ultrasound also showed aortic arch wall thickening in several groups, but LLC NPs maintained smoother vascular walls, highlighting their potential therapeutic benefits against atherosclerosis.

### Analysis of pathological indexes of atherosclerotic plaque

**Fig. 5:**
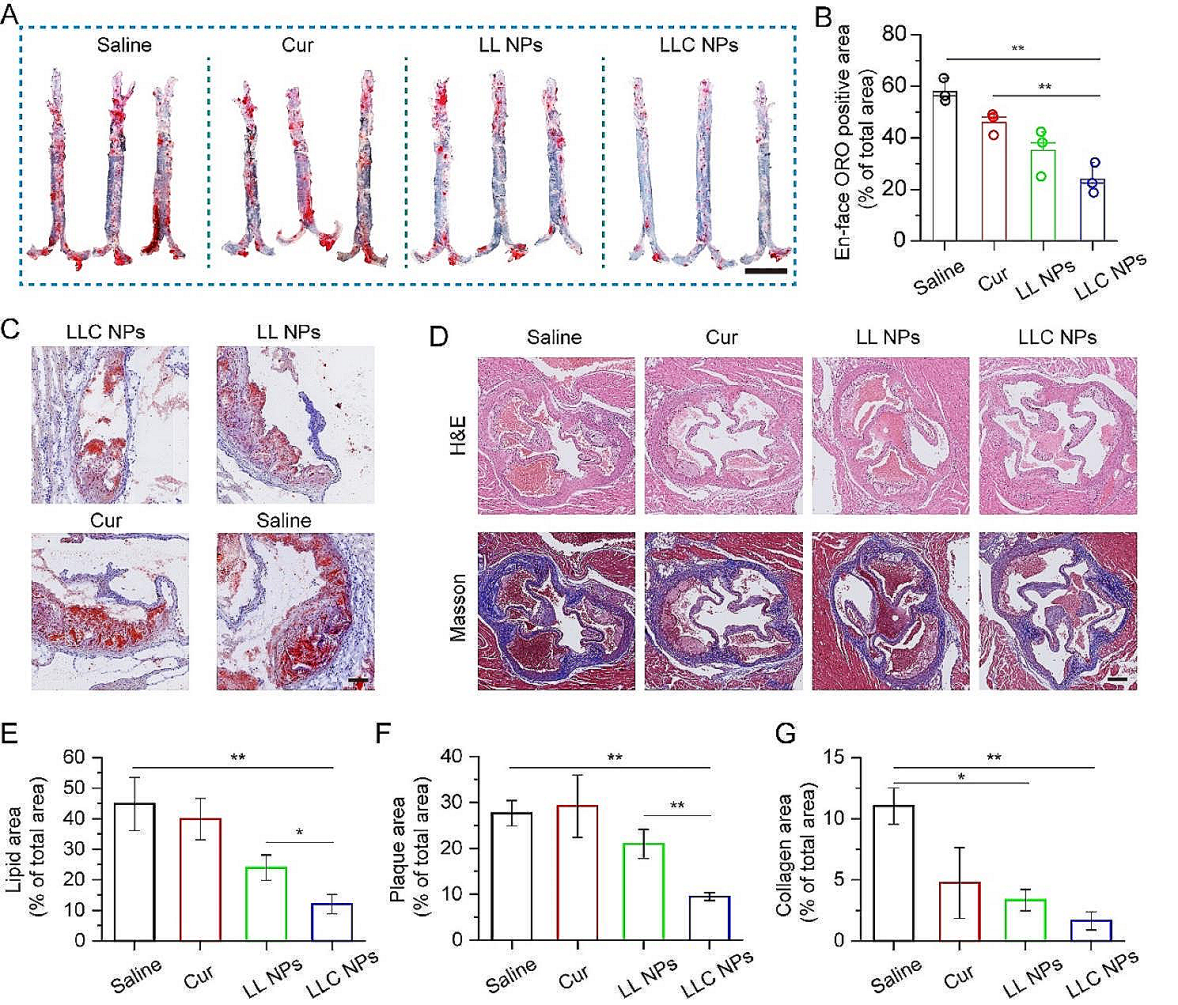
Assessment of the therapeutic effect of NPs on arterial plaque base on histological staining. (**A**) Image of ORO-stained en face aortas (Scale bar = 5 mm). (**B**) Measurements of areas staining positive for ORO in aortas subjected to various treatments. Mean ± SD, *n* = 3, **p < 0.01. (**C**) Representative images of aortic sinus stained with ORO (Scale bar = 150 μm). (**D**) Exemplary images of sections from the aortic sinus, highlighted by H&E and Masson staining (Scale bar = 250 μm). (**E**-**G**) Quantitative proportion of plaque in aortic sinus section samples from ORO (**C**), H&E and Masson (**D**) staining. Mean ± SD, *n* = 3, *p < 0.05, **p < 0.01

In light of positive targeting effect and imaging evaluation, analysis of pathological indexes of atherosclerotic plaque was furtherly conducted. Following the completion of treatment, the entire aorta was carefully extracted and the Oil Red O (ORO) staining of aorta was performed. As shown in Fig. 5A, a large ORO-positive area could be found in isolated aorta of apoe^−/−^ mice treated with saline, and both Cur and LL NPs demonstrated a modest reduction in plaque formation, due to the respective anti-inflammation and anti-monocyte adhesion. In contrast, LLC NPs demonstrated remarkable efficacy in combating atherosclerosis, owing to its excellent ability to target arterial plaques and cascading anti-inflammatory effects. The analysis of ORO-stained areas within the aorta (Fig. 5B) revealed that the therapeutic efficacy of LLC NPs greatly surpassed that of free Cur. In addition, sections of aortic sinus were also stained with ORO for analysis of lipid content in plaques. As shown in Fig. 5C, the red area represented the lipids and the lipid content in the plaques of mice treated with LLC NPs was significantly lower than that of other groups, which was consistent with the results of the ORO staining of aorta. Furthermore, to analyze the rate of luminal stenosis and the collagen content within plaques, the H&E and Masson staining of aortic sinus sections was conducted. As shown in Fig. 5D, there were obvious lipid pools in the plaques of mice in the saline group, but only foam cells were found on the vascular wall of mice in the treatment group, which indicated that LLC NPs could significantly alleviate the process of atherosclerosis. Besides, collagen had penetrated into the plaques of mice in the saline group, while there was no significant collagen generation in the plaques of other treatment groups. We further quantitatively calculated the luminal stenosis rate and collagen content in the aortic sinus, and found that LLC NPs could significantly reduce the luminal stenosis rate and collagen content in the plaque, further indicating the excellent therapeutic effect of LLC NPs (Fig. 5E-G).

### Mechanism of anti-atherosclerosis action and safety evaluation

**Fig. 6:**
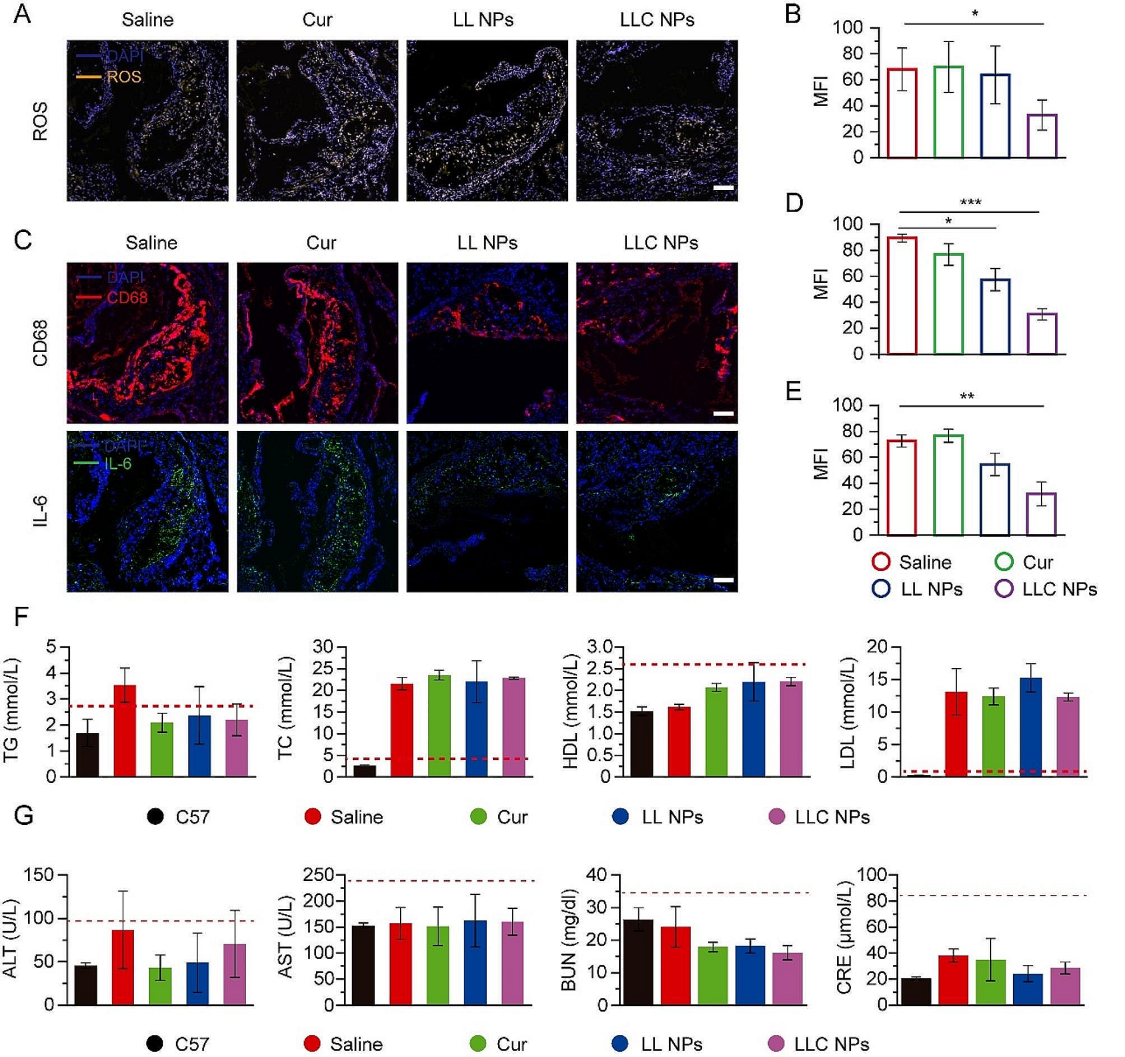
Pathological analysis and serum analysis. (**A**) Fluorescent images of aortic sinus stained with dihydroethidium (DHE) and DAPI from atherosclerosis mice (Scale bar = 100 μm). (**B**) Quantitative analysis of ROS positive area from (**A**). Mean ± SD, *n* = 3, *p < 0.05. (**C**) Sections of aortic sinus stained with antibody to CD68, IL-6 (Scale bar = 100 μm). (**D**, **E**) Quantitative analysis of CD68 positive and IL-6 positive area from (**C**). Mean ± SD, *n* = 3, *p < 0.05, **p < 0.01, ***p < 0.0001. (**F**) The serum level of TG, TC, HDL and LDL in all groups. Mean ± SD, *n* = 4. (**G**) Indices of hepatic and renal functions after treatment in apoe^−/−^ mice. Means ± SD, *n* = 3

Based on the good anti-atherosclerosis effect of LLC NPs in vivo, we further analyzed the biological indicators within plaque to study the therapeutic mechanism. Firstly, the ROS within the plaque of aortic sinus was investigated. As shown in Fig. 6A, yellow fluorescence represented regions with high expression of ROS, which were concentrated inside the plaque. Compared with the saline group, the yellow fluorescence in plaques of LLC NPs group was significantly weakened, showing a good anti-ROS effect, which was attributed to ROS responsive drug release and antioxidant activity of Cur (Fig. 6B). ROS mainly originated from the secretion of macrophages in plaques, so the content of macrophages in plaques was also investigated. In Fig. 6C, red fluorescence represented macrophages, which were widely distributed on the surface and inside of plaques in saline group. Free Cur had almost no therapeutic effect due to its poor water solubility. The NPs treatment group showed low and weak red fluorescence due to its inhibition of monocyte adhesion, thereby reducing the content of macrophages in plaques, and their effect were significantly better than that of the saline group (Fig. 6D). Moreover, IL-6, as a representative inflammatory factor, was also detected. The green fluorescence represented IL-6, which was also distributed inside the plaque. Consistent with macrophage distribution, IL-6 in plaque was obviously reduced in LLC NPs group (Fig. 6E).

Blood lipid was an important index to detect the course of atherosclerosis. Therefore, the lipid content in the blood of mice were recorded. We found that the total cholesterol (TC) and low density lipoprotein (LDL) levels in the blood of the atherosclerotic mice were much higher than those of the healthy mice. However, there was no significant difference in blood lipids indicators, including TC, triglycerides (TG), LDL, and high-density lipoprotein (HDL), among the treatment groups (Fig. 6F). The above results indicated that the anti-atherosclerosis mechanism of LLC NPs was mainly related to the cascade inhibition of ROS and anti-inflammation, and had nothing to do with lipid regulation. For safety evaluation, liver and kidney function indicators were detected in Fig. 6G. There were not obvious differences in ALT, AST, BUN and CRE among groups. No obvious damage was observed in the H&E stained sections of the liver, spleen, and kidneys (Fig. S7). Besides, Body weight of mice remained consistent across all groups during the treatment, suggesting that LMWH-LA as drug delivery system had a good biosafety profile (Fig. S8).

## Conclusion

In summary, we had developed LLC NPs with cascading inhibition of ROS in plaque for atherosclerosis treatment. The carrier components LMWH-LA conjugates were synthesized through a simple esterification reaction. Curcumin was encapsulated in the hydrophobic core of the stable, biocompatible micelles with the ability to target atherosclerotic plaques. As LLC NPs accumulated within plaques, LMWH bound to P-selectin on plaque endothelial cells, thus blocking the pathway of monocytes entering the interior of the plaque to lead to decreased content of inflammatory cells that can produce ROS. In addition, LA played a role in clearing ROS and achieved a transition from hydrophobicity to hydrophilicity to accelerate the release of curcumin, further alleviating oxidative stress and inhibiting inflammation. In mice with atherosclerosis, ultrasound imaging provided a detailed assessment of the abdominal aorta and aortic arch post-treatment. Findings indicated that LLC NPs significantly reduced oxidative stress and curbed the progression of atherosclerosis, all without causing systemic toxicity. Therefore, the developed LMWH-LA micelles could be a promising candidates for atherosclerosis therapy.

## Experimental section

### Materials

Low molecular weight heparin (LMWH, MW 3800 ∼ 5000) was purchased from Melonepharma (Dalian, China). Alphalipoic acid (LA, 99%), 1,1-diphenyl-2-picrylhydrazyl (DPPH), N, N-dimethylformamide (DMF), formamide, 1-[3-(dimethylamino) propyl]-3-ethylcarbodiimide hydrochloride (EDC) (> 99%), N-hydroxy-succinimide (NHS) (> 98%) and (dimethylamino)pyridine (DMAP) (> 99%) was obtained from Macklin (Shanghai, China). 3-(4,5-dimethyl-2-tiazolyl)-2,5-diphenyl-2Htetrazolium bromide (MTT) and Hoechst 33342 were obtained from Beyotime Institute of Biotechnology (Shanghai, China). Anti-CD68 and Anti-IL6 primary antibodies were purchased form Abcam (Shanghai, China). Lipopolysaccharide (LPS), Cyanine5.5 (Cy5.5), and 2’, 7’-dichlorodihydrofluorescein diacetate (DCFH-DA) were from Sigma-Aldrich (St. Louis, MO). Other reagents and chemicals were of reagent grade.

### Cell lines and animals

Human Umbilical Vein Endothelial Cells (HUVEC), RAW264.7 cells, and THP-1 cells were supplied by the Institute of Biochemistry and Cell Biology at the Chinese Academy of Sciences in Shanghai, China. Additionally, C57BL/6 and apoe-/- male mice, aged approximately 6 weeks and weighing between 18 and 22 g, were acquired from the Model Animal Research Center at Nanjing University, located in Nanjing, China. All animal experiments were approved and performed following the guidelines of the Animal Care and Use Committee of Donghua University (approval # DHUEC-STCSM-2023-01) and also in accordance with the policy of the National Institute of Health (China). The apoe^−/−^ mice were fed with a high-fat diet for 4 weeks to construct the atherosclerosis model. Establishment of atherosclerosis model was verified by ultrasound imaging and pathology method.

### Synthesis and characterization of LMWH-LA

The amphiphilic conjugate was synthesized by bonding the hydrophilic segment LMWH to the hydrophobic LA unit via an ester bond. Initially, LA (200 mg) along with EDC (372 mg), NHS (223 mg), and DMAP (59 mg) were mixed in N, N-dimethylformamide (19 mL) and stirred under nitrogen gas at room temperature for 3 h to activate LA’s carboxylic acid groups. Subsequently, LMWH (200 mg), pre-dissolved in formamide (8 mL) at 50 °C, was gradually introduced to the activated LA. This mixture then reacted for 48 h at room temperature under nitrogen, followed by dialysis (MWCO 3500) to remove the organic solvent and impurities, and freeze-drying to obtain pure LMWH-LA. The synthesis was verified using [1]H NMR, Fourier transform infrared (FTIR) spectra, and thermogravimetric analysis (TGA).

### Preparation and characterization of NPs

The conjugate (10 mg) was mixed into a CH_2_Cl_2_ solution (1 mL), then introduced to water (5mL) with ultrasonication (2s/2s, 300w, 5 min) to create an emulsion, which was rotary evaporated to remove CH_2_Cl_2_ to produce LL NPs. For LLC NPs, a CH_2_Cl_2_ solution (1 mL) containing 1 mg of curcumin and 10 mg of LMWH-LA conjugate to 5 mL of water and performed ultrasonic emulsification. The resulting LLC NPs solution was obtained after rotary evaporation and filtration using a 0.45 μm filter. The size and zeta potentials of the NPs were analyzed utilizing a Malvern Zetasizer Nano ZS90 (Worcestershire, UK). The shapes and structures of the nanoparticles were examined using a transmission electron microscope (TEM, JEOL JEM-1400Plus, Tokyo, Japan). The stability of NPs was assessed by monitoring the variations in their size and polydispersity index (PDI) over time on the method of dynamic light scattering (DLS) through the Malvern Zetasizer Nano ZS90 at 4 °C. The NPs were combined with 50% fetal bovine serum (FBS) and kept at 37 °C. Their transparency was then measured at a wavelength of 750 nm using a UV-visible spectrophotometer. (PerkinElmer, Boston, MA) at different time points. The critical micelle concentration (CMC) of NPs was determined using DLS. Meanwhile, the release behavior of Cur from the LLC NPs was explored at 37 °C in a phosphate-buffered saline (PBS) solution with a pH of 7.4, utilizing a UV-visible spectrophotometer for analysis.

### Electronic supplementary material

Below is the link to the electronic supplementary material.

Supplementary Material 1

## Data Availability

No datasets were generated or analysed during the current study.

## References

[1] Rohatgi A. Stressing the endothelium to assess localized inflammatory potential and the risk for atherosclerotic Cardiovascular Disease. Circulation. 2021;143(20):1946–8. 33999663 10.1161/CIRCULATIONAHA.121.053989 PMC8162315

[2] Lu X, He Z, Xiao X, Wei X, Song X, Zhang S. Natural antioxidant-based Nanodrug for Atherosclerosis Treatment. Small. 2023;n/a(n/a):2303459. 10.1002/smll.202303459 37607320

[3] Moore KJ, Sheedy FJ, Fisher EA. Macrophages in atherosclerosis: a dynamic balance. Nat Rev Immunol. 2013;13(10):709–21. 23995626 10.1038/nri3520 PMC4357520

[4] He J, Zhang W, Zhou X, Xu F, Zou J, Zhang Q, Zhao Y, He H, Yang H, Liu J. Reactive oxygen species (ROS)-responsive size-reducible nanoassemblies for deeper atherosclerotic plaque penetration and enhanced macrophage-targeted drug delivery. Bioactive Mater. 2023;19:115–26. 10.1016/j.bioactmat.2022.03.041 PMC9010555 35475030

[5] He Y, Liu T. Oxidized low-density lipoprotein regulates macrophage polarization in atherosclerosis. Int Immunopharmacol. 2023;120:110338. 37210916 10.1016/j.intimp.2023.110338

[6] Jung HW, Ra M, Bae HJ, Hong S-P. The LDL-C/Apo B predicts coronary atherosclerotic heart disease in non-diabetic patients without high LDL-C. Medicine 2023, *102* (1). 10.1097/MD.0000000000032596 PMC9829249 36607865

[7] Navia-Pelaez JM, Agatisa-Boyle C, Choi S-H, Sak Kim Y, Li S, Alekseeva E, Weldy K, Miller YI. Differential expression of inflammarafts in Macrophage Foam cells and in Nonfoamy macrophages in atherosclerotic lesions—brief report. Arterioscler Thromb Vasc Biol. 2023;43(2):323–9. 36453276 10.1161/ATVBAHA.122.318006 PMC9877149

[8] Yu X-H, Fu Y-C, Zhang D-W, Yin K, Tang C-K. Foam cells in atherosclerosis. Clin Chim Acta. 2013;424:245–52. 23782937 10.1016/j.cca.2013.06.006

[9] Jaipersad Anthony S, Lip Gregory YH, Silverman S, Shantsila E. The role of monocytes in Angiogenesis and Atherosclerosis. J Am Coll Cardiol. 2014;63(1):1–11. 24140662 10.1016/j.jacc.2013.09.019

[10] Langlois M, Duprez D, Delanghe J, De Buyzere M, Clement DL. Serum vitamin C concentration is low in peripheral arterial disease and is Associated with inflammation and severity of atherosclerosis. Circulation. 2001;103(14):1863–8. 11294804 10.1161/01.CIR.103.14.1863

[11] Salonen RM, Nyyssönen K, Kaikkonen J, Porkkala-Sarataho E, Voutilainen S, Rissanen TH, Tuomainen T-P, Valkonen V-P, Ristonmaa U, Lakka H-M, Vanharanta M, Salonen JT, Poulsen HE. Six-year effect of combined vitamin C and E supplementation on atherosclerotic progression. Circulation. 2003;107(7):947–53. 12600905 10.1161/01.CIR.0000050626.25057.51

[12] Upston JM, Kritharides L, Stocker R. The role of vitamin E in atherosclerosis. Prog Lipid Res. 2003;42(5):405–22. 12814643 10.1016/S0163-7827(03)00024-9

[13] Hodis HN, Mack WJ, LaBree L, Mahrer PR, Sevanian A, Liu C-r, Liu C-h, Hwang J, Selzer RH, Azen SP. Alpha-tocopherol supplementation in healthy individuals reduces low-density lipoprotein oxidation but not atherosclerosis. Circulation. 2002;106(12):1453–9. 12234947 10.1161/01.CIR.0000029092.99946.08

[14] Ma X, Zhang T, Luo Z, Li X, Lin M, Li R, Du P, Yu X, Ma C, Yan P, Su J, Wang L, Li Y, Jiang J. Functional nano-vector boost anti-atherosclerosis efficacy of berberine in Apoe(–/–) mice. Acta Pharm Sinica B. 2020;10(9):1769–83. 10.1016/j.apsb.2020.03.005 PMC7564017 33088695

[15] Ramji DP. Polyunsaturated fatty acids and atherosclerosis: insights from Pre-clinical studies. Eur J Lipid Sci Technol. 2019;121(1):1800029. 10.1002/ejlt.201800029

[16] Wang Q, Duan Y, Jing H, Wu Z, Tian Y, Gong K, Guo Q, Zhang J, Sun Y, Li Z, Duan Y. Inhibition of atherosclerosis progression by modular micelles. J Controlled Release. 2023;354:294–304. 10.1016/j.jconrel.2023.01.020 36638843

[17] Santhakumar AB, Battino M, Alvarez-Suarez JM. Dietary polyphenols: structures, bioavailability and protective effects against atherosclerosis. Food Chem Toxicol. 2018;113:49–65. 29360556 10.1016/j.fct.2018.01.022

[18] Cheng Y-C, Sheen J-M, Hu WL, Hung Y-C. Polyphenols and Oxidative Stress in Atherosclerosis-Related Ischemic Heart Disease and Stroke. *Oxidative Medicine and Cellular Longevity* 2017, *2017*, 8526438. 10.1155/2017/8526438 PMC5727797 29317985

[19] Sugamura K, Keaney JF. Reactive oxygen species in cardiovascular disease. Free Radic Biol Med. 2011;51(5):978–92. 21627987 10.1016/j.freeradbiomed.2011.05.004 PMC3156326

[20] Wang Y, Li L, Zhao W, Dou Y, An H, Tao H, Xu X, Jia Y, Lu S, Zhang J, Hu H. Targeted therapy of atherosclerosis by a broad-spectrum reactive oxygen species scavenging nanoparticle with intrinsic anti-inflammatory activity. ACS Nano. 2018;12(9):8943–60. 30114351 10.1021/acsnano.8b02037

[21] Kornfeld OS, Hwang S, Disatnik M-H, Chen C-H, Qvit N, Mochly-Rosen D. Mitochondrial reactive oxygen species at the heart of the Matter. Circul Res. 2015;116(11):1783–99. 10.1161/CIRCRESAHA.116.305432 PMC4443500 25999419

[22] Mecocci P, Polidori MC. Antioxidant clinical trials in mild cognitive impairment and Alzheimer’s disease. Biochim et Biophys Acta (BBA) - Mol Basis Disease. 2012;1822(5):631–8. 10.1016/j.bbadis.2011.10.006 22019723

[23] Li Z, Wang Q, Jing H, Luo X, Du L, Duan Y. cRGD peptide-modified nanocarriers for targeted delivery of angiogenesis inhibitors to Attenuate Advanced atherosclerosis. ACS Appl Nano Mater. 2021;4(11):11554–62. 10.1021/acsanm.1c02009

[24] Gao C, Huang Q, Liu C, Kwong CHT, Yue L, Wan J-B, Lee SMY, Wang R. Treatment of atherosclerosis by macrophage-biomimetic nanoparticles via targeted pharmacotherapy and sequestration of proinflammatory cytokines. Nat Commun. 2020;11(1):2622. 32457361 10.1038/s41467-020-16439-7 PMC7251120

[25] Chen W, Schilperoort M, Cao Y, Shi J, Tabas I, Tao W. Macrophage-targeted nanomedicine for the diagnosis and treatment of atherosclerosis. Nat Reviews Cardiol. 2022;19(4):228–49. 10.1038/s41569-021-00629-x PMC8580169 34759324

[26] Prilepskii AY, Serov NS, Kladko DV, Vinogradov VV. Nanoparticle-Based Approaches towards the Treatment of Atherosclerosis *Pharmaceutics* [Online], 2020. 10.3390/pharmaceutics12111056 PMC7694323 33167402

[27] Feng X, Xu W, Li Z, Song W, Ding J, Chen X, Nanosystems I. Adv Sci. 2019;6(17):1900101. 10.1002/advs.201900101 PMC6724480 31508270

[28] Qu S, Liu R, Zhang N, Xu Y, Yue X, Dai Z. Non-viral nucleic acid therapeutics: revolutionizing the landscape of atherosclerotic treatment. Nano Today. 2022;45:101514. 10.1016/j.nantod.2022.101514

[29] Ma B, Xu H, Zhuang W, Wang Y, Li G, Wang Y. Reactive oxygen species responsive theranostic nanoplatform for two-Photon Aggregation-Induced Emission Imaging and Therapy of Acute and chronic inflammation. ACS Nano. 2020;14(5):5862–73. 32379416 10.1021/acsnano.0c01012

[30] Cai M, Cao J, Wu Z, Cheng F, Chen Y, Luo X. In vitro and in vivo anti-tumor efficiency comparison of phosphorylcholine micelles with PEG micelles. Colloids Surf B. 2017;157:268–79. 10.1016/j.colsurfb.2017.05.053 28601755

[31] Wang Q, Lei D, Chen F, Chen Y, Luo X. Tracing difference: in Vitro and in Vivo Antitumor Property Comparison of pH-Sensitive Biomimetic Phosphorylcholine Micelles with Insensitive Micelles. ACS Biomaterials Sci Eng. 2019;5(5):2258–70. 10.1021/acsbiomaterials.9b00027 33405777

[32] Liu W, Zhang Y, Wei G, Zhang M, Li T, Liu Q, Zhou Z, Du Y, Wei H. Integrated Cascade Nanozymes with Antisenescence activities for Atherosclerosis Therapy. Angew Chem Int Ed 2023, 62 (33), e202304465. 10.1002/anie.202304465 37338457

[33] Wang Q, Jing H, Lin J, Wu Z, Tian Y, Gong K, Guo Q, Yang X, Wang L, Li Z, Duan Y. Programmed prodrug breaking the feedback regulation of P-selectin in plaque inflammation for atherosclerotic therapy. Biomaterials. 2022;288:121705. 36002347 10.1016/j.biomaterials.2022.121705

[34] Mahmoudi-Nezhad M, Vajdi M, Farhangi MA. An updated systematic review and dose-response meta-analysis of the effects of α-lipoic acid supplementation on glycemic markers in adults. Nutrition. 2021;82:111041. 33199187 10.1016/j.nut.2020.111041

