# Supplementary material for: Targeted nanoparticles triggered by plaque microenvironment for atherosclerosis treatment through cascade effects of reactive oxygen species scavenging and anti-inflammation
Source: J Nanobiotechnology. 2024 Jul 27;22:440. doi: 10.1186/s12951-024-02652-9 (PMC11282716; doi:10.1186/s12951-024-02652-9)
Supplement: Supplementary file 1 — Supplementary Material 1 [file 12951_2024_2652_MOESM1_ESM.docx]

Targeted nanoparticles triggered by plaque microenvironment for atherosclerosis treatment through cascade effects of reactive oxygen species scavenging and anti-inflammation

Xianghong Luo^1, #^, Mengjiao Zhang^2, 3, #^, Waicong Dai^4^, Xianghao Xiao^4^, Xinyi Li^3^, Yingjian Zhu^5, *^, Xiangyang Shi^4, *^, Zhaojun Li^3, 6, *^

^1^ Department of Echocardiography, Shanghai General Hospital, Shanghai Jiaotong University School of Medicine, Shanghai 200080, China.

^2^ Department of Medical Imaging, Weifang Medical University, Weifang 261053, Shandong, China

^3^ Department of Ultrasound, Shanghai General Hospital, Shanghai Jiaotong University School of Medicine, Shanghai 200080, China

^4^ State Key Laboratory for Modification of Chemical Fibers and Polymer Materials, Shanghai Engineering Research Center of Nano-Biomaterials and Regenerative Medicine, College of Biological Science and Medical Engineering, Donghua University, Shanghai 201620, China

^5^ Department of Urology, Jiading Branch of Shanghai General Hospital, Shanghai Jiao Tong University School of Medicine, Shanghai 201803, China

^6^ Department of Ultrasound, Jiading Branch of Shanghai General Hospital, Shanghai Jiaotong University School of Medicine, Shanghai 201803, China

*Corresponding authors: Zhaojun Li; E-mail: [lzj_1975@sina.com](mailto:lzj_1975@sina.com)

Xiangyang Shi; E-mail: xshi@dhu.edu.cn

Yingjian Zhu; E-mail: zhuyingjian_sjtu@126.com

^#^Xianghong Luo and Mengjiao Zhang contributed equally to this work.

Catalog

[1. Experimental Section 2](#_Toc167951005)

[1.1 Materials 2](#_Toc167951006)

[1.2 Cell lines and animals 3](#_Toc167951007)

[1.3 Synthesis and characterization of LMWH-LA 3](#_Toc167951008)

[1.4. Preparation and characterization of NPs 3](#_Toc167951009)

[1.5 In vitro hemolysis assay 4](#_Toc167951010)

[1.6 Radical scavenging activity of NPs 4](#_Toc167951011)

[1.7 In vitro drug release 5](#_Toc167951012)

[1.8 In vitro cytotoxicity analysis 5](#_Toc167951013)

[1.9 Cellular uptake in RAW264.7 cells 5](#_Toc167951014)

[1.10 Detection of effect of NPs on the adhesion between THP-1 cells and HUVECs in vitro 6](#_Toc167951015)

[1.11 ROS and inflammatory factors detection in RAW264.7 cells 6](#_Toc167951016)

[1.12 In vivo accumulation of NPs 6](#_Toc167951017)

[1.13 In vivo anti-atherosclerosis assay 6](#_Toc167951018)

[1.14. In vivo toxicology evaluations 7](#_Toc167951019)

[1.15 Statistical analysis 7](#_Toc167951020)

[2. Synthesis and characterization of LMWH-uFA 7](#_Toc167951021)

[3. Particle size of LL NPs 8](#_Toc167951022)

[4. The morphology of red blood cells 9](#_Toc167951023)

[5. Cell viability 9](#_Toc167951024)

[6. H&E Staining of liver, spleen, and kidneys 9](#_Toc167951025)

[7. Body weights 10](#_Toc167951026)

# 1. Experimental Section

## 1.1 Materials

Low molecular weight heparin (LMWH, MW 3800~5000) was purchased from Melonepharma (Dalian, China). Alphalipoic acid (LA, 99%), 1,1-Diphenyl-2-picrylhydrazyl (DPPH), N, N-Dimethylformamide (DMF), formamide, 1-[3-(dimethylamino) propyl]-3-ethylcarbodiimide hydrochloride (EDC) (> 99%), N-Hydroxy-succinimide (NHS) (> 98%) and (Dimethylamino)pyridine (DMAP) (> 99%) was obtained from Macklin (Shanghai, China). 3-(4,5-dimethyl-2-tiazolyl)-2,5-diphenyl-2Htetrazolium bromide (MTT), Hoechst 33342 were obtained from Beyotime Institute of Biotechnology (Jiangsu, China). Anti-CD68, Anti-IL6 primary antibody were purchased form Abcam (Shanghai, China). Lipopolysaccharide (LPS), Cyanine5.5 (Cy5.5), 2′,7′-Dichlorodihydrofluorescein diacetate (DCFH-DA) were purchased from Sigma Aldrich (USA). Other reagents and chemicals were analytical level.

## 1.2 Cell lines and animals

Human Umbilical Vein Endothelial Cells (HUVEC), RAW264.7 cells, THP-1 cells were provided by the Institute of Biochemistry and Cell Biology at the Chinese Academy of Sciences (Shanghai, China). C57BL/6 male mice and apoe^-/-^ male mice (about 6-week-old, 18-22 g) were purchased from Model Animal Research Center of Nanjing University (Nanjing, China). All animal experiments were approved and performed following the guidelines of the Animal Care and Use Committee of Donghua University and also in accordance with the policy of the National Institute of Health (China). The apoe^-/-^ mice were fed a high-fat diet for 4 weeks to construct the atherosclerosis model. Establishment of atherosclerosis model was verified by ultrasound imaging and pathology method.

## 1.3 Synthesis and characterization of LMWH-LA

The hydrophilic segment LMWH was bonded to the hydrophobic unit LA through an ester bond to form an amphiphilic conjugate. First, LA, EDC, NHS, and DMAP were dissolved in N, N-dimethylformamide under N_2_ at room temperature. The mixture was stirred for 3 h to activate the carboxylic acid groups in LA. Then, LMWH was dissolved in formamide at 50 °C and added slowly to the above activated LA solution. The mixture was reacted for 48 h under N_2_ at room temperature and then transferred into a dialysis bag (MWCO 3500) to remove organic solvent and impurities. Finally, pure LMWH-LA was obtained through vacuum freeze drying. The successful synthesis of LMWH-LA was confirmed by ^1^H-NMR, Fourier transform infrared (FTIR) spectra, and thermogravimetric analysis (TGA).

## 1.4. Preparation and characterization of NPs

LMWH-LA was dispersed into a dichloromethane solution, and the mixture was added to deionized water under ultrasonication (2s/2s, 300w, 5 min). After the obtained emulsion was rotary evaporated to remove CH_2_Cl_2_, LL NPs was obtained. For LLC NPs, For LLC NPs, a CH_2_Cl_2_ solution (1 mL) containing 1 mg of curcumin and 10 mg of LMWH-LA conjugate to 5 mL of water and performed ultrasonic emulsification. The resulting LLC NPs solution was obtained after rotary evaporation and filtration using a 0.45 μm filter. The size and zeta potentials of the NPs were measured by dynamic light scattering (DLS) using a Malvern Zetasizer Nano ZS90 (Malvern, U.K.). The morphologies of the NPs were observed under a transmission electron microscope (TEM, JEOL JEM-1400Plus). To evaluate the stability of the micelles, the changes in micelle size and PDI with time were investigated by DLS at 4 °C. NPs were mixed with 50% FBS and incubated at 37 °C. The transmittance of the sample was measured at 750 nm using an ultraviolet‒visible spectrophotometer (PerkinElmer, Boston, MA) at different times. The critical micelle concentration (CMC) of NPs was measured with the light scattering method. The release profiles of Cur from LLC NPs were investigated at 37 °C in PBS (pH 7.4) by ultraviolet-visible spectrophotometer.

## 1.5 In vitro hemolysis assay

10% red blood cell (RBC) suspension was prepared from C57BL/6 mice. LL NPs with different concentrations were incubated with RBC suspension for 1 h at 37 °C. The incubation solution was centrifuged at 5000 r/min for 4 min, and the supernatant was taken to measure the absorbance (OD) value of 540 nm with a Varioskan Flash multimode reader (Thermo, USA). Pure water was set as a positive control, and 0.9% saline was as a negative control. The hemolysis rate was calculated according to the following formula:

Hemolysis (%) = (OD_sample_ - OD_negative_ / (OD _positive_ - OD _negative_)) × 100%.

## 1.6 Radical scavenging activity of NPs

First, a 180 μM DPPH solution in absolute ethanol was prepared and stored in the dark. Then 1.0 mL of the sample aqueous solution at different concentration (0.5, 1, 2, and 4 mg/mL) was mixed with 2.0 mL of DPPH-ethanol solution and then incubated in the dark for 20 min at room temperature. Meanwhile, the DPPH solution was substituted with absolute ethanol as the blank, while deionized water was used instead of the sample aqueous solution as the control. Subsequently, the decrease of DPPH radicals was determined by measuring the decrease of absorbance at 517 nm. The assay was performed in triplicate for all the samples and the measured values are calculated by the following formula to obtain the DPPH radical scavenging rate of the test samples.

Scavenging effect (%) = [1 − (As − Ab)/Ac] × 100

Where As is the absorbance of the sample at 517 nm, Ab is the absorbance of the blank group and Ac is the absorbance of the control group at 517 nm.

## 1.7 In vitro drug release

The release profiles of Cur from LLC NPs were investigated at 37 °C in PBS (pH 7.4), and set the concentration of H_2_O_2_ in the medium to 0, 0.1 mM and 1 mM. Briefly, 1 mg/mL LLC NPs (3 mL) were added into dialysis bags (MWCO 3500), then the dialysis bags were immersed in 200 mL PBS. At desired time intervals, 100 μL was taken out from dialysis bags and the content of released Cur was determined by ultraviolet-visible spectrophotometer (AOE UV-9000, China).

## 1.8 In vitro cytotoxicity analysis

Cytotoxicity of formulations was investigated using RAW264.7 cells. RAW264.7 cells were inoculated in 96-well plates and when reached up to about 60% confluence, different concentrations of LL NPs were added. After 24 h of incubation, MTT (5 mg/mL, 20 μL per well) was added and incubated for 4 h. Then, RAW264.7 cells were washed with PBS, and DMSO (150 μL per well) was added and incubated for 15 min at 37 ^o^C. The absorbance of each well was measured by microplate reader at 490 nm. The calculation formula of cell viability was as follows.

Cell viability (%) = [(Abs)_test_ – (Abs)_Blank_]/[(Abs)_control_ – (Abs)_Blank_] ⅹ100%

## 1.9 Cellular uptake in RAW264.7 cells

In order to investigate the cellular uptake of LLC NPs, fluorescence microscope (Olympus IX51, Olympus Corporation, Japan) and flow cytometer (Becton Dickinson Facscan analyzer, Franklin Lakes, NJ) were used. Briefly, RAW264.7 cells were seeded in 6-well plates (3 × 10^5^ per well) and cultured at 37 °C in a 5% CO_2_ atmosphere overnight. After incubation with LLC NPs (concentration of Cur was 5 μg/mL) for 1 h and 4 h, the culture medium was replaced with Hoechst 33342 solution (5 μg/mL) and incubated for 15 min. Finally, the cells were washed with PBS several times and observed using fluorescence microscope. In addition, RAW264.7 cells (1 × 10^5^ cells per well) were incubated with LLC NPs for 1 h and 4 h. After trypsinization, the cells were collected in 0.3 mL PBS and tested by flow cytometry.

## 1.10 Detection of effect of NPs on the adhesion between THP-1 cells and HUVECs in vitro

Activated HUVECs were pretreated with LMWH, LL NPs for 1 h. Then the green fluorescent labeled THP-1 cells were added and incubated with HUVECs for another 30 min at 37 °C. The culture supernatant was removed and HUVECs were washed to remove unadhered THP-1 cells. Fluorescence microscope (Olympus IX51, Olympus Corporation, Japan) was used to observe the adhesion condition, and the number of cells in each field is statistically analyzed. Inactivated HUVECs was taken as control group.

## 1.11 ROS and inflammatory factors detection in RAW264.7 cells

RAW264.7 cells were treated with PBS, LPS, Cur + LPS, LL NPs + LPS, and LLC NPs + LPS for 24 h. ROS generation in RAW264.7 cells were also detected with DCFH-DA by fluorescence microscope and flow cytometry. The culture supernatant was collected and the TNF-α, IL-6, CRP were measured by enzyme-linked immunosorbent assay (ELISA) (Neobioscience, China).

## 1.12 In vivo accumulation of NPs

Cy5.5 labeled LL NPs was intravenously injected to atherosclerosis mice. After 24 h, the mice were sacrificed. The aortas, heart, liver, spleen, lung, and kidney were isolated. Ex vivo imaging was performed, and the fluorescent intensity was measured by IVIS spectrum system (PerkinElmer, USA).

## 1.13 In vivo anti-atherosclerosis assay

After the successful establishment of the atherosclerosis model, the apoe^-/-^ male mice were randomly divided into 4 groups (n = 7), and the treatment was started on the 5th week after high fat diet. The mice were intravenously injected with saline, Cur, LL NPs, and LLC NPs (at an equivalent dose of 3 mg/kg Cur. w/w). Each formulation was administered twice a week for 8 weeks. C57BL/6 mice with normal diet acted as a negative control group. After treatment, ultrasound imaging was performed *in vivo* to detect the aortic arch and abdominal aorta in mice to evaluate the therapeutic effect. Then, the mice were euthanized and their aortas were separated, opened longitudinally and stained with ORO. Sections of the aorta were prepared and stained with ORO, H&E and Masson. For immunohistochemistry study, sections of aortic tissue were incubated with antibodies to CD68, IL-6, and α-smooth muscle actin (α-SMA). In addition, serum was collected for blood lipid analysis.

## 1.14. In vivo toxicology evaluations

During treatment, the body weight of all mice was recorded once a week. At the end of treatment, the mice were sacrificed, and the major organs (heart, liver, spleen, lung and kidney) were isolated. The histological sections were prepared and stained with H&E to evaluate the toxicity of NPs. Moreover, whole blood was obtained in anticoagulative tubes, and the serum was collected for liver and kidney functions analysis.

## 1.15 Statistical analysis

Data was reported as mean ± SD. All statistical analyses were performed using SPSS version 21.0 (SPSS Inc., Chicago, IL, USA). The experimental data were statistically analyzed by using the t-test between two independent samples. A p-value < 0.05 was considered as significantly different. P value less than 0.05, 0.01, 0.001, and 0.0001 were denoted by *, **, *** and ****, respectively.

# 2. Synthesis and characterization of LMWH-uFA


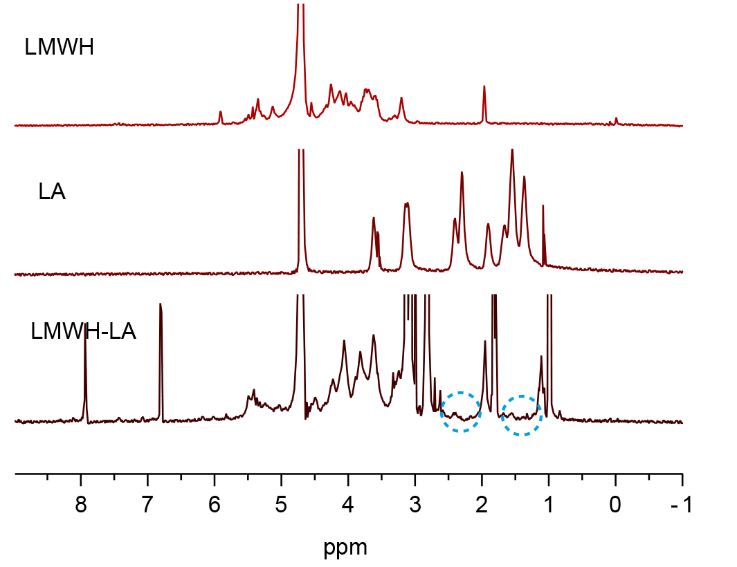


Fig. S 1 ^1^H NMR spectrum of LMWH, LA and LMWH-LA in D_2_O.


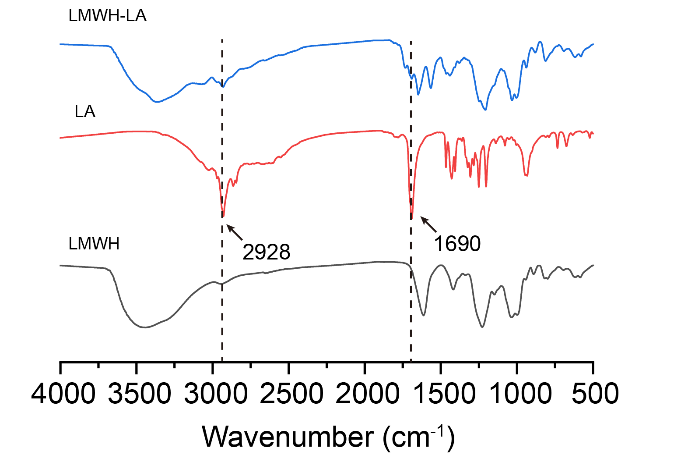


Fig. S 2 FT-IR spectra of LMWH, LA and LMWH-LA. The IR characteristic peak of LA appeared in the IR spectrum of LMWH-LA, indicating LA conjugated to the LMWH chain.


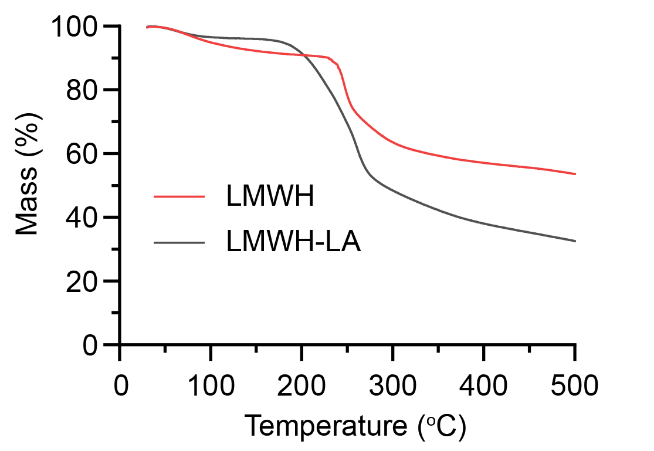


Fig. S 3 TGA curves of LMWH and LMWH-LA.

# 3. Particle size of LL NPs


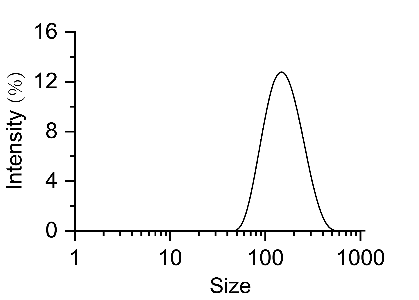


Fig. S 4 Particle size of LL NPs.

# 4. The morphology of red blood cells


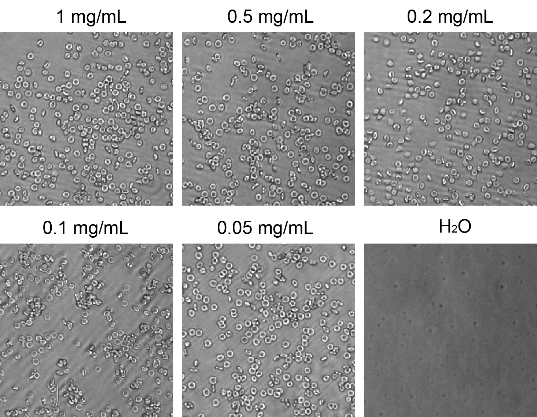


Fig. S 5 The morphology of red blood cells cultured with LL NPs at various concentrations.

# 5. Cell viability


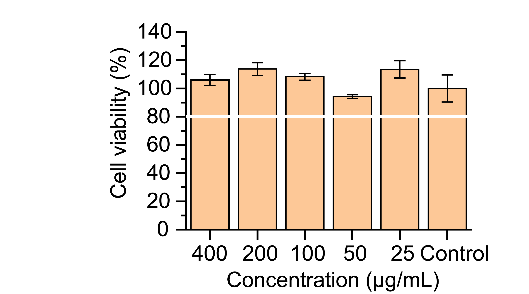


Fig. S 6 Cell viability of RAW264.7 cells with LL NPs for 24 h.

# 6. H&E Staining of liver, spleen, and kidneys


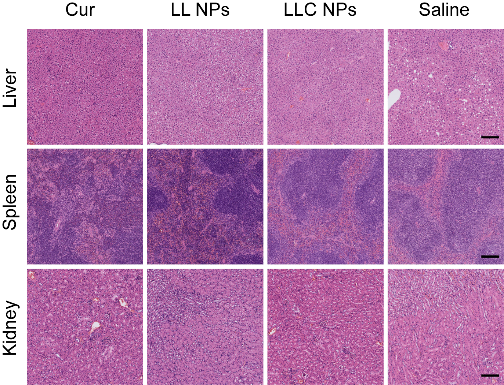


Fig. S 7 Representative liver, spleen and kidney sections stained with H&E (scale bar = 200 μm).

# 7. Body weights


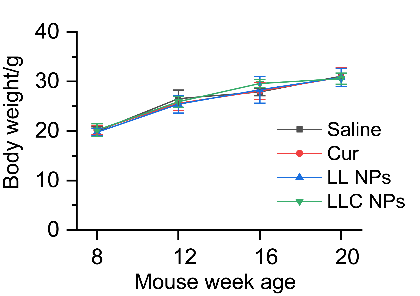


Fig. S 8 The body weights of the mice during the treatment (means ± SD, n = 5).
